# Acute effects of nonexcitatory electrical stimulation during systole in isolated cardiac myocytes and perfused heart

**DOI:** 10.14814/phy2.12106

**Published:** 2014-08-05

**Authors:** Ksenia Blinova, Jayna Stohlman, Victor Krauthamer, Alan Knapton, Erik Bloomquist, Richard A. Gray

**Affiliations:** 1 Center for Devices and Radiological Health, US Food and Drug Administration, Silver Spring, Maryland, USA; 2 Center for Drug Evaluation and Research, US Food and Drug Administration, Silver Spring, Maryland, USA

**Keywords:** Calcium, cardiac contractility modulation, cardiac electrical stimulation, cardiomyocytes, fluo‐4, optical imaging

## Abstract

Application of electrical field to the heart during the refractory period of the beat has been shown to increase the force of contraction both in animal models and in heart failure patients (cardiac contractility modulation, or CCM). A direct increase in intracellular calcium during CCM has been suggested to be the mechanism behind the positive inotropic effect of CCM. We studied the effect of CCM on isolated rabbit cardiomyocytes and perfused whole rat hearts. The effect of CCM was observed in single cells via fluorescent measurements of intracellular calcium concentration ([Ca^2+^]_i_) and cell length (L). Cells were paced once per second throughout these recordings, and CCM stimulation was delivered via biphasic electric fields of 20 ms duration applied during the refractory period. CCM increased the peak amplitude of both [Ca^2+^]_i_ and L for the first beat during CCM compared to control, but then [Ca^2+^]_i_ and L decayed to levels lower than the control. During CCM, all contractions had a faster time to peak for both [Ca^2+^]_i_ and L; after stopping CCM the rise times returned to control levels. In the whole rat heart, the positive inotropic effect of CCM stimulation on left ventricular pressure was completely abolished in the presence of metoprolol, a beta‐1 adrenergic blocker. In summary, the CCM‐induced changes in intracellular calcium handling by cardiomyocytes did not explain the sustained positive inotropic effect in the whole heart and the *β*‐adrenergic pathway may be involved in the CCM mechanism of action.

## Abstract (precis)

Cardiac contractility modulation (CCM) is a heart failure therapy which delivers electrical pulses to the heart during refractory period. While there are some promising reports on the therapy's safety and effectiveness in humans, the underlining mechanism remains unknown. We studied the effect of CCM pulses in isolated rabbit cardiomyocytes and isolated rat heart in the presence of beta adrenergic blocker and recorded intracellular calcium transients and contractions. We concluded that the CCM‐induced changes in intracellular calcium handling by cardiomyocytes did not explain the sustained positive iotropic efect in the whole heart and beta‐adrenergic pathway may be involved in the CCM mechanism of action.

## Introduction

Heart failure (HF), which affects 5.1 million Americans, is associated with poor quality of life and reduction in life expectancy (Go et al. 2013). Systolic HF is characterized by severe impairment of cardiac contractile function corresponding to a decreased ejection fraction in patients. Attempts to improve cardiac contractility through the use of inotropic pharmacological agents can provide short‐term improvements in contractile function and quality‐of‐life factors, but lead to a significant increase in patient mortality (Stevenson 1998). More recent conventional drug therapies (i.e., *β*‐adrenoceptor blockers, diuretics, angiotensin converting enzyme inhibitors) alleviate some symptoms and reduce mortality rates. However, it is recognized that patients on these drugs continue to deteriorate over time. The success of cardiac resynchronization therapy (CRT) indicates that electrical therapy is a complementary treatment for HF patients with an increased QRS duration.

An investigational alternate electrical therapy for HF, cardiac contractility modulation (CCM), has been suggested. CCM entails the application of an electrical field during systole and the absolute refractory period of the contractile cycle and hence is referred to as “non‐excitatory” electrical therapy (Burkhoff et al. 2001; Burkhoff and Ben‐Haim 2005; Imai et al. 2007). Randomized clinical trial results reported that CCM did not have an adverse effect on hospitalization or mortality, or improved the primary endpoint of ventilatory anaerobic threshold, but it did improve peak oxygen consumption (VO_2_) assessed via cardiopulmonary stress test, and lower the Minnesota Living with HF Questionnaire score (Borggrefe et al. 2008; Kadish et al. 2011). Clinical trials from smaller cohorts of patients reported that CCM did not increase myocardial oxygen consumption (unlike cAMP‐dependent positive ionotropic drugs) (Butter et al. 2007), may normalize expression of genes for sarcoplasmic reticulum calcium cycling (Butter et al. 2008), and improved global and regional LV contractility as measured using tissue Doppler imaging (Yu et al. 2009). In situ CCM experiments on dogs show similar results (Imai et al. 2007; Gupta et al. 2009a,b). Reported CCM effects included: LV pressure increase in isolated ferret and rabbit hearts (Mohri et al. 2003; Winter et al. 2011); a polarity‐dependent effect on force in rabbit papillary and human trabecular muscle tissues (Mohri et al. 2002; Brunckhorst et al. 2006); and an increase in cell shortening in isolated myocytes (Sabbah et al. 2001). In addition, an increase in calcium delivery into cardiac myocytes due to modulation of the action potential has been proposed as a mechanism of the CCM inotropic effect (Burkhoff et al. 2001).

In order to asses CCM therapy in various patient populations and its potential interactions with other therapies (e.g., pharmaceuticals (Krauthamer and Smith 2004)), it is important to know how the electrical fields corresponding to CCM affect myocyte dynamics, particularly regarding inotopicity as regulated by [Ca^2+^]_i_. For example, if CCM increases myocyte force development, the cellular effect could enhance sympathetic effects; however, if CCM decreases myocyte force development, the overall effect of CCM may be suboptimal and could be altered to minimize the deleterious cellular behavior. Therefore, we studied the acute effects of CCM on isolated myocytes and isolated perfused hearts. We present results on CCM electrical stimulation effect on isolated cardiac myocytes and isolated heart, and discuss possible mechanism of action for these effects.

## Materials and Methods

### Isolation of cardiac myocytes

Cardiac myocytes were isolated from adult male rabbit hearts using standard procedures (Chacon et al. 1994) with minor modifications. Briefly, white New Zealand rabbits weighing 3–4 kg were heparinized by administration of 250 USP units of sodium heparin per kg of body weight via marginal ear vein injection. Acepromazine was administered by the same route for sedation. Isoflurane was used for deep inhalational anesthesia. The chest cavity was opened, the heart removed, and immediately placed in an ice‐cold Ca^2+^‐free Buffer A (5 mmol/L KCI, 110 mmol/L NaCl, 1.2 mmol/L NaH_2_PO_4_, 28 mmol/L NaHCO_3_, 30 mmol/L glucose, 0.05 units/mL insulin, 250 *μ*mol/L adenosine, 1 mmol/L creatine, 1 mmol/L carnitine, 1 mmol/L taurine, 10 units/mL penicillin, 10 units/mL streptomycin, 20 mmol/L butanedione monoxime, 250 *μ*mol/L adenosine, 1 mmol/L octanoic acid, and 25 mmol/L HEPES, pH 7.3). Each heart was mounted on a modified Langendorff perfusion apparatus and perfused at a rate of 25 mL/min with Buffer A containing 1 mmol/L EGTA and 2 USP units/mL of sodium heparin saturated with 95% O_2_, 5% CO_2_ in a retrograde fashion at 37°C from a height of 100 cm. After 5 min, a digestion buffer consisting of Buffer A containing 25 *μ*mol/L CaCl_2_, 68 units/mL collagenase type II, and 70 units/mL hyaluronidase was recirculated for 20 min. Using scissors, the ventricles were excised below the atrioventricular junction, and four incisions toward the apex were made. Using sterile forceps, the tissue was then gently agitated in Buffer A supplemented with 25 *μ*mol/L CaCl_2_ and 0.5 mg/mL trypsin 1:250 for 30 min at room temperature to release the rod‐shaped myocytes. Cells were centrifuged for 5 min at 25G and resuspended in cell culture media at room temperature consisting of a 1:1 mixture of Joklik's medium and medium 199 supplemented with 20 mmol/L butanedione monoxime, 1 mmol/L creatine, 1 mmol/L carnitine, 1 mmol/L taurine, 0.05 units/mL insulin, 10 units/mL penicillin, and 10 mg/mL streptomycin at pH 7.4. Cells were finally washed three times with the same cell culture media without butanedione monoxime and stored in a 37°C, 5% CO_2_ incubator. Cells were resuspended in an imaging buffer (20 mmol/L HEPES, 10 mmol/L glucose, 120 mmol/L NaCl, 4.7 mmol/L KCl, 1 mmol/L KH_2_PO_4_, 1 mmol/L MgSO_4_, 1 mmol/L creatine, 1 mmol/L taurine, 20 mmol/L NaHCO_3_, 5 mmol/L sodium lactate, and 1 mmol/L CaCl_2_) for all fluorescent imaging experiments.

### Isolated cardiac myocytes: Fluorescence measurements and image analysis

Isolated cardiac myocytes were stained with Fluo‐4, AM, or di‐8‐ANEPPS (Life Technologies, Grand Island, NY) for intracellular calcium ([Ca^2+^]_i_) and cell length (L) measurements, correspondingly. Isolated myocytes were placed in a 236 *μ*L imaging chamber (Warner Instruments, Hamden, CT) and superfused with the imaging buffer containing either 2 *μ*mol/L fluo–4, AM for 20 min or 1.25 *μ*mol/L di‐8‐ANEPPS for 6 min at room temperature. After staining, cells were continuously superfused with the pure imaging buffer at the rate of 3–4 mL/min, and the temperature of the perfusion buffer and the chamber was gradually raised to 37°C.

**Figure 1.**
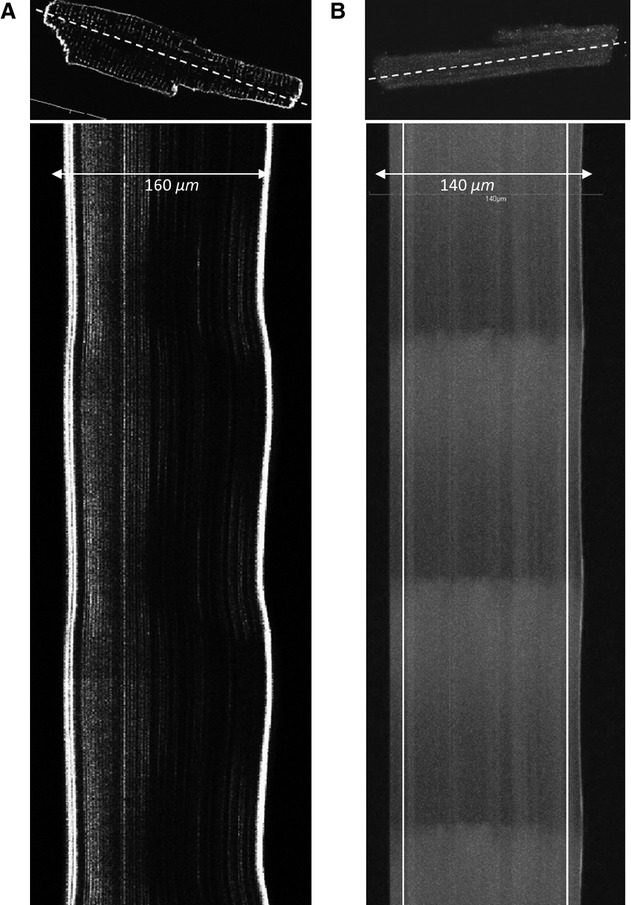
Optical imaging of single cardiomyocytes. Fluorescent image of single cardiomyocyte stained with di‐8 ANEPPS for cell length measurements (A, top panel) and fluo‐4, AM for intracellular calcium ([Ca^2+^]_i_) measurements (B, top panel). Cardiomyocytes were scanned along the long axis of the cell (dotted line) and time series were collected (bottom panel). Cell length was calculated as a distance between the brightly stained edges of the cell (A). For the [Ca^2+^]_i_ calculations, fluorescence intensity was averaged for each time line in between white vertical lines, omitting the edges of the cell (B).

Fluorescent imaging was performed by acquiring line scans on a confocal microscope (FluoView1000, Olympus, Center Valley, PA) with an Uplan Apochromat 1.40 numerical aperture 40 × oil immersion objective and fully opened pinhole (800 *μ*m diameter). The scan line was oriented along the long axis of the cardiomyocyte and took 2–3 ms to collect. About 10,000–25,000 line scans were obtained from each cell (up to 60 sec) and processed using the Java‐based program ImageJ (Rasband 1997). Fluorescent time series of cardiomyocytes stained with either fluo‐4, AM or di‐8‐ANEPPS are shown in Figure 1. The cell length was measured using di‐8‐ANEPPS staining of the cell membranes, and calculating the distance between the edges of the cell along the scan line (Fig. 1A). For the calcium transients, plots of the average fluo‐4, AM fluorescence intensity was calculated for each line scan with the edges of the cell excluded from the analysis (white lines on the graph, Fig. 1B).

### Whole perfused heart experiments

Male Sprague–Dawley rats weighing 300–375 g were used. Rats were anesthetized with isoflurane and given 100 USP units sodium heparin intravenously. After removal, hearts were immediately placed in ice‐cold oxygenated Buffer B (118 mmol/L NaCl, 5 mmol/L KCl, 1.2 mmol/L MgSO_4_, 25 mmol/L NaHCO_3_, 1.25 mmol/L CaCl_2_, 11 mmol/L glucose, pH 7.4). Hearts were cannulated through the aorta and suspended in a chamber warmed by 37°C circulating water. Hearts were perfused in a retrograde manner at a constant pressure of 60 mmHg with 37°C Buffer B saturated with a mixture of 95% O_2_ and 5% CO_2_. A latex balloon connected to a Harvard Apparatus pressure transducer was inserted into the left ventricle and inflated with distilled water. Left ventricular pressure parameters were recorded continuously. Hearts were allowed to stabilize for 30 min prior to treatment.

### Electrical field (CCM) stimulation

The cardiomyocyte imaging chamber was equipped with a pair of platinum wire electrodes attached to the parallel sides of the oval‐shaped bath. Both pacing and CCM electrical pulses were delivered through this pair of electrodes, resulting in field stimulation of the isolated rabbit cells. For whole rat heart experiments, both stimuli were delivered through a concentric bipolar electrode, in direct contact with the ventricular epicardium. Square wave electrical pulses (monophasic) were generated using LabView software and signal strength was controlled and amplified through constant current stimulators (Model A385, World Precision Instruments, Sarasota, FL). Single cells and whole hearts were continuously paced at 1 Hz (2 ms stimulus pulse duration) and CCM stimulation was delivered as four 5 ms biphasic pulses (20 ms total duration). The delay between pacing pulses and CCM stimulation was 30 ms in single rabbit cardiomyocyte experiments, the same as in the CCM devices in the clinical practice. Action potential duration in the rat heart is significantly shorter than in rabbit (see for example (Szigligeti et al. 1996)), so we decreased the delay between the pacing pulse and CCM stimulation to 10 ms in the whole rat heart experiments to stay within the refractory period and avoid potential arrhythmias. Pacing and CCM stimuli amplitudes were optimized to obtain consistent cardiac response and maximize the CCM effect on left ventricular pressure in the whole heart, or on the cells length changes in the isolated cardiomyocytes. In whole heart experiments, the optimal pacing current was 8 mA and for CCM pulses, 17 mA. Field potentials in the imaging chamber for the single‐cell measurements were set at 10 V/cm for pacing and 5 V/cm for CCM stimulation. Responses from continuously paced cells and whole hearts were compared before, during, and after CCM stimulation as described in the Results section.

### Statistics

Using normalized signals for calcium and length, values for peak, duration and time to peak, a linear mixed effects model was fit using SAS software version 9.2 (Cary, NC). For duration and time to peak measurements, the model used a linear slope before, during, and after CCM, and allowed for a discontinuous jump at the beginning of each stage of the experiment (before, during, and after CCM). For the peak calcium measurement, a linear slope was used before CCM and after CCM, but during CCM, an exponential decay fit was used. For the peak length measurement, we allowed for a linear slope before CCM and after CCM, as well as an exponential growth fit during CCM. The exponential growth fit for the CCM portion of the peak length measurement started with the second beat of CCM. For all three measurements, the error structure of the model was assumed to be a one‐step autoregressive process with homogeneous residual errors.

## Results

### Isolated myocytes: Calcium

**Figure 2.**
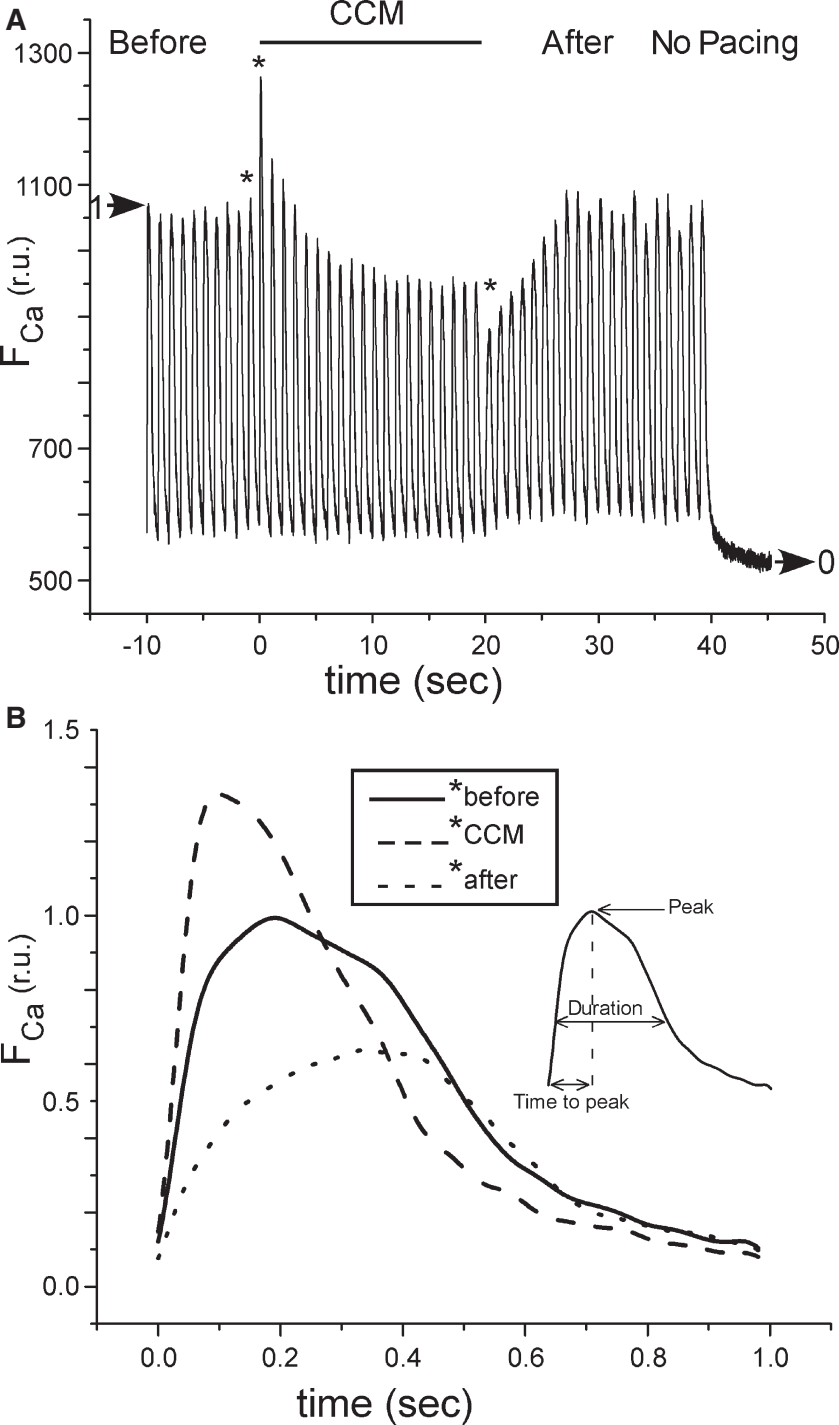
Effect of CCM on relative intracellular calcium ([Ca^2+^]_i_). (A) An example of a raw calcium signal (duration of 55 sec) indicating the time course of [Ca^2+^]_i_ before, during, and after CCM followed by an interval with no pacing. The large horizontal arrows indicate the normalization procedure; all signals were normalized between 0 and 1. (B) The time course of normalized [Ca^2+^]_i_ for three beats indicated by asterisks in (A). The inset indicates the quantification of the [Ca^2+^]_i_ transients in Figure 3.

**Figure 3.**
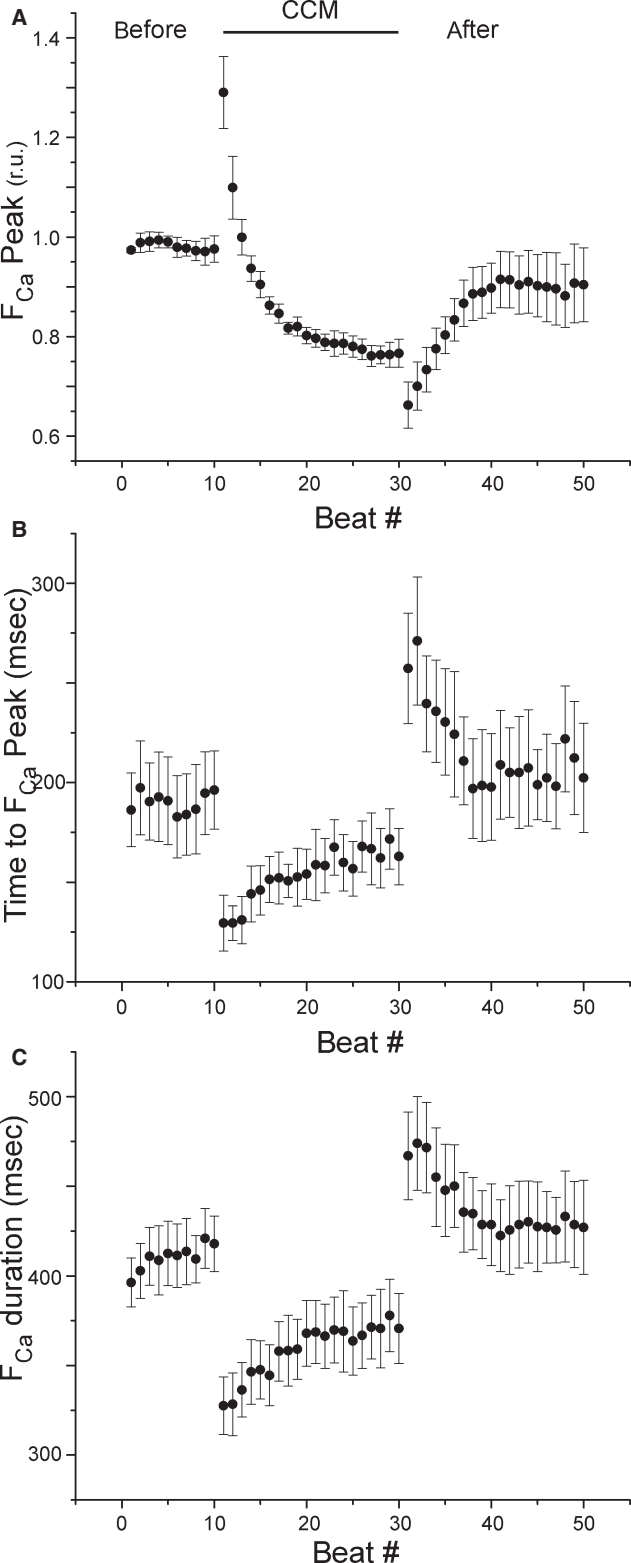
Quantification of beat‐to‐beat effects of CCM on [Ca^2+^]_i_. The beat‐to‐beat dynamics of peak [Ca^2+^]_i_ (A), time to peak [Ca^2+^]_i_ (B), and duration of [Ca^2+^]_i_ transient (C) before (beats 0–9), during (beats 10–29), and after (beats 30–49) CCM.

A representative fluorescence calcium signal transient, [Ca^2+^]_i_, is shown at the top of Figure 2 before, during and after CCM (time zero was defined as the start of CCM). The raw signals were normalized such that the average magnitude of the [Ca^2+^]_i_ signal from 44–45 sec was set to zero and the peak amplitude of the first beat (*t* = −10 sec) was set to one. The effect of CCM on [Ca^2+^]_i_ transients was quantified by analyzing the peak amplitude, time to peak, and duration (measured at 50% peak amplitude) for each beat from the six cells, which were isolated from six different rabbits (see Fig. 2B, inset, and Statistical Methods). The average and standard error of the mean are shown for these three variables for all beats in Figure 3. None of these three variables exhibited a significant positive or negative slope in the nine beats prior to CCM, but all variables were affected by CCM. For the first beat immediately after the start of CCM, the peak [Ca^2+^]_i_ amplitude increased by 32.4% (*P *<**0.05); the time to peak decreased by 33.1% (*P *<**0.05); and the [Ca^2+^]_i_ transient duration decreased by 28.1% (*P *<**0.05). During CCM, the peak amplitude decreased in a nonlinear manner to a level significantly below (16.1%, *P *<**0.05) control, that is, there was a difference between the last beat before CCM and the last beat during CCM. During CCM, the time to peak did not exhibit a significant slope but the duration did (with a slope of 0.0038 per beat, *P *<**0.05). For the first beat after CCM was turned off, the peak amplitude immediately decreased by 12.7% (*P *<**0.05); the time to peak immediately increased by 58.2% (*P *<**0.05); and the duration immediately increased by 31.4% (*P *<**0.05). After the cessation of CCM, at the conclusion of the experiment, the peak, time to peak, and duration all returned to [Ca^2+^]_i_ levels found at the start of the experiment, that is, there was no difference between the first beat and the last beat of each recording (peak *P*‐value = 0.43, duration *P*‐value = 0.54, time to peak *P*‐value = 0.99).

### Isolated myocytes: Length

**Figure 4.**
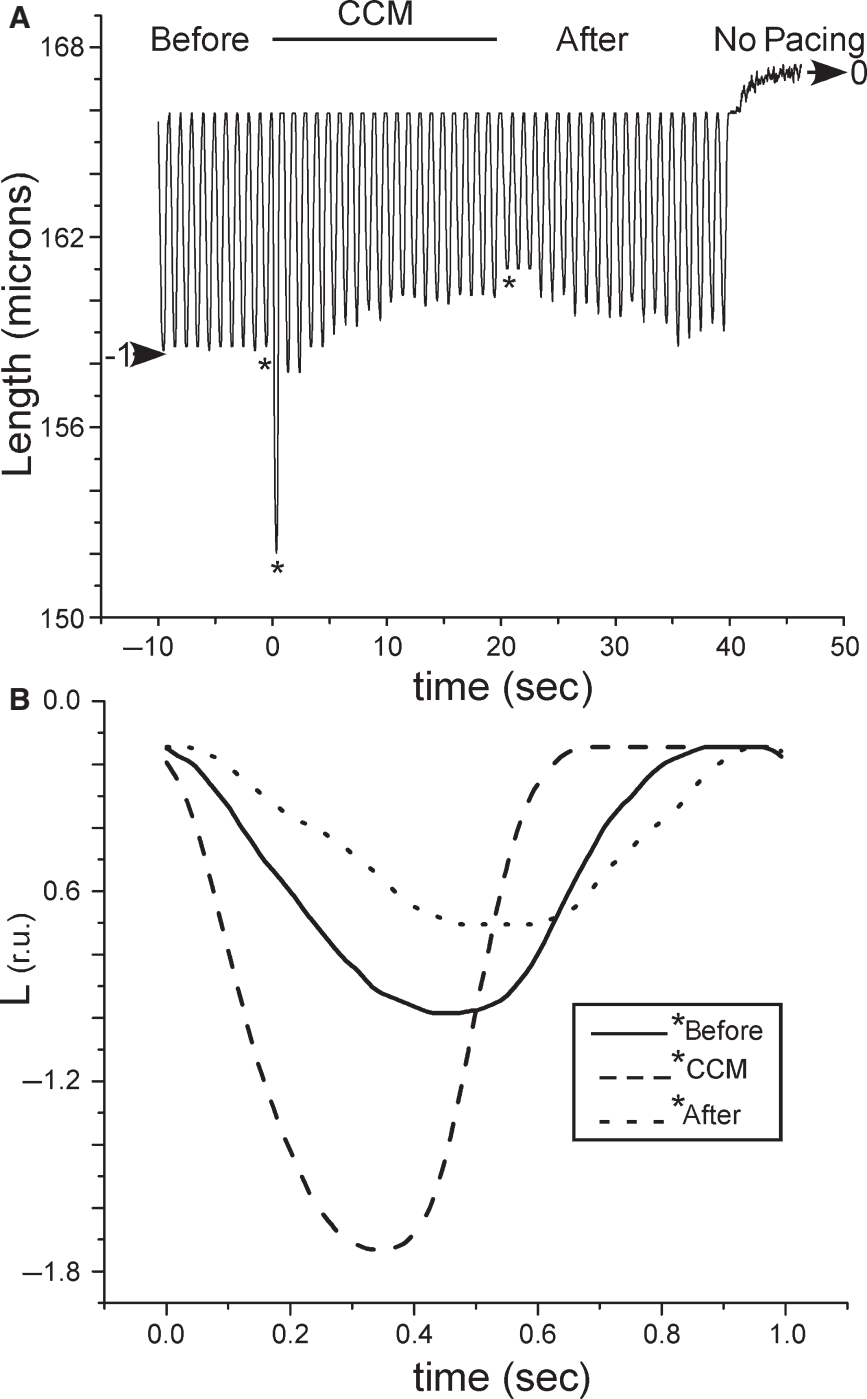
Effect of CCM on myocyte length. (A) An example of a raw cell length signal (duration of 55 sec) indicating the time course of length before, during, and after CCM followed by an interval with no pacing. The large horizontal arrows indicate the normalization procedure. (B) The time course of normalized length (L) for three beats indicated by asterisks in (A). Length was normalized as for [Ca^2+^]_i_ (see inset in Fig.****2)

**Figure 5.**
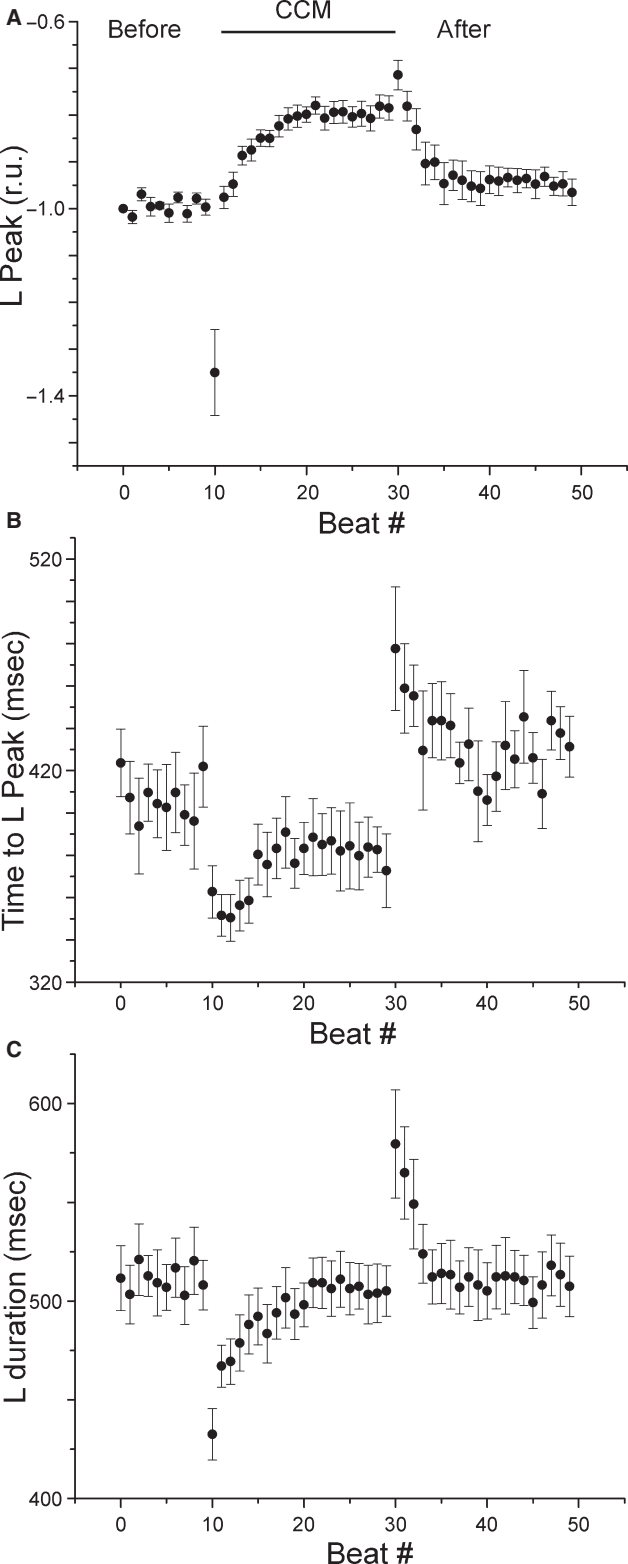
Quantification of beat‐to‐beat effects of CCM on length (L). The beat‐to‐beat dynamics of peak L (A), time to peak L (B), and duration of L transient (C) before (beats 0–9), during (beats 10–29), and after (beats 30–49) CCM.

A representative cell length transient before, during, and after CCM is shown at the top of Figure 4. Length was analyzed in the same way as the calcium transient (peak amplitude, time to peak, and duration) as shown in Figure 4B. The raw signals were normalized such that the length from 44–45 sec was set to zero and the peak amplitude of the first beat was set to negative one. The effect of CCM on cell shortening was quantified by analyzing the peak, time to peak, and duration for each beat from the same six cells as for calcium. The average and standard error of the mean are shown for these three variables for all beats in Figure 5. None of the three variables exhibited a significant slope in the nine beats prior to CCM but all variables were affected by CCM. For the first beat immediately after the start of CCM, cell shortening increased by 35.6% (*P *<**0.05); the time to peak increased by 12.3% (*P *<**0.05); and the duration decreased by 14.2% (*P *<**0.05). During CCM after the first beat, the peak amplitude decreased in an exponential manner to a level significantly smaller than control (19.8%, *P *<**0.05) and the time to peak decreased linearly to a level nearly significantly different than control (8.9%, *P *=**0.058). The previous estimates are the differences between the last beat immediately before CCM and the last beat during CCM. After the first beat of CCM, the length duration exhibited a significant slope of 4.0 ms per beat, *P *<**0.05. For the first beat after CCM was turned off, the peak amplitude immediately decreased by 3.6% (*P *<**0.16); the time to peak immediately increased by 22.8% (*P *<**0.05); and the length duration immediately increased by 9.0% (*P *<**0.05). At the conclusion of the experiment, the peak, time to peak, and duration all returned to levels found at the start of the experiment (peak *P*‐value = 0.82, duration *P*‐value = 0.33, time to peak *P*‐value = 0.76).

Beta‐1‐blockers, metoprolol (2 *μ*mol/L), and atenolol (20 *μ*mol/L) did not significantly alter CCM effects on [Ca^2+^]_i_ transient amplitude, duration, or cell shortening in isolated cardiac myocytes (data not shown).

### Heart: Pressure

**Figure 6.**
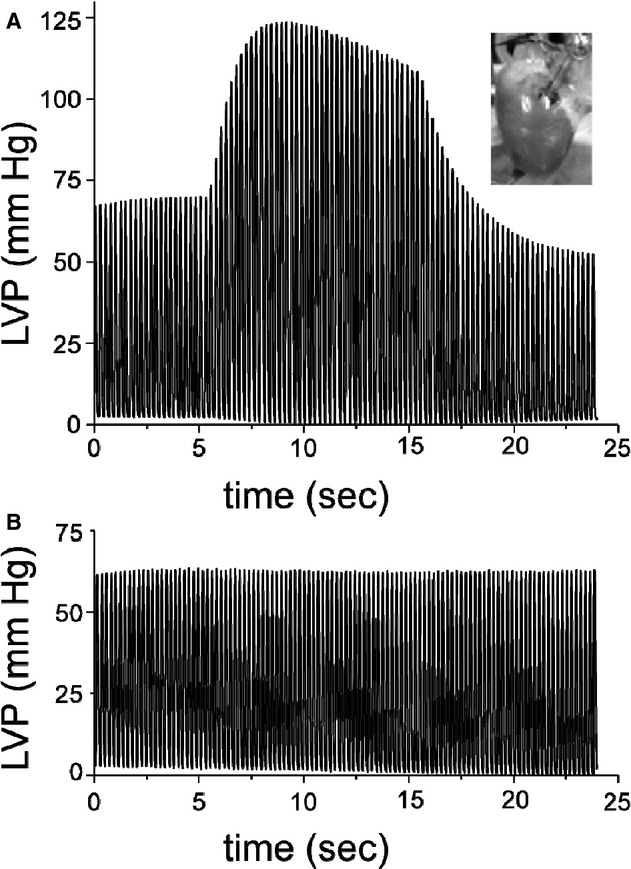
Cardiac contractility modulation effect in a whole heart is abolished in the presence of *β*1‐adrenoceptor antagonist metoprolol. Left ventricular pressure was measured in a whole rat heart being paced at 4 Hz. Ten seconds of CCM stimulation was applied in addition to the pacing from 6 to 16 sec of the pacing protocol in both experiments (A and B). In the first experiment, (A) the heart was perfused with control buffer, and in the second experiment (B) a bolus injection of 1 mL of 1 mmol/L metoprolol was performed into the perfusion media right before data collection.

In the whole rat heart, CCM resulted in an immediate increase in left ventricular pressure (LVP) as shown in Figure 6A (which confirms previously reported results, *add appropriate reference*) and this inotropic effect of CCM was completely abolished in the presence of *β*1‐adrenoceptor antagonist metoprolol (Fig. 6B). The top graph shows the effect of 10 sec of electrical modulation pulses delivered with a 10 ms CCM delay after each S1 pulse on the left ventricular pressure in the perfused rat heart. The bottom graph shows the block of CCM effect on LVP following a bolus injection of 1 mL of 1 mmol/L metoprolol into the perfusion media. The effect of metoprolol was completely reversible, and the CCM inotropic effect was observed at the baseline level 15 min after the wash‐out (data not shown).

In isolated cardiomyocytes, where beta receptor agonist release is absent, CCM did not induce a sustained increase in contractility or intracellular calcium transients. These data suggest that the positive inotropic effect of CCM in the whole heart is not by direct action on cardiomyocytes. Blocking of CCM effect by metoprolol in the whole heart suggests that *β*‐adrenergic pathway is likely to be involved in the CCM mechanism of action.

## Discussion

Despite the suggestion that CCM increases contractility via an increase in intracellular calcium concentration (Burkhoff et al. 2001; Lyon et al. 2013), there is very little evidence of this mechanism of action (Sabbah et al. 2001; Mohri et al. 2003). The only research to date on CCM effect in isolated cardiomyocytes (Rasband 1997) reports a 27% increase in the extent of shortening and a 19% increase in both peak and integral calcium transients. One limitation of this study is that their data represent averaged values over the first 10 beats during CCM. Our study is the first to look at beat‐to‐beat changes in cardiomyocyte function during CCM. Our results show that there is an increase in both cell contraction and intracellular calcium transient amplitude in the first few beats immediately after CCM stimulation is turned on, but after the first several beats this positive inotropic effect diminishes and the consequent contractions are actually weaker than at the baseline.

Identifying the mechanisms involved in clinical CCM is very difficult because; not only are the effects of nonuniform electric fields throughout the heart very complex, but the possible means of nonexcitatory electric fields directly affecting LVP are limited. It has been previously shown in whole hearts that the increase in LVP induced by CCM is abolished by beta adrenergic blockers consistent with the mode of action of CCM involving myocardial *β*1‐adrenoceptors stimulation (Kapa et al. 2010; Winter et al. 2011). Our results from the whole rat hearts support this idea. However, until our study, the effect of CCM nonexcitatory electric fields on isolated myocytes was unknown. By examining the effect upon single isolate myocytes, we are able to demonstrate a seemingly contradictory direct effect in which CCM diminishes systolic [Ca^2+^]_i_ and cell shortening.

Specifically, we found an immediate effect of CCM such that the largest effect occurred during the beat for which CCM was initiated: peak [Ca^2+^]_i_ increased while its time to peak and duration decreased (see Figs. 2–3); cell shortening increased while its time to peak and duration decreased (see Figs. 4–5). This immediate effect of CCM strongly implies an effect on the L‐type calcium current and/or the sodium‐calcium exchanger. However, the immediate effects of CCM decreased to lower than the control levels after only 20 sec of the electrical “therapy”. After cessation of CCM, there was a brief “rebound” effect, but all quantities returned to control levels within 20 sec.

The main limitation of this study is that we studied the acute effects of CCM on healthy isolated rabbit myocytes and did not consider important molecular and pathway changes within cardiomyocytes that occur in chronic heart failure. It would also be important to further investigate CCM mechanism in a heart with chronic failure in addition to the healthy cardiac models used here.

Our results suggest that the mode of action of CCM involves both direct effects upon [Ca^2+^]_i_ and indirect effects upon the cardiac beta‐adrenergic efferents. Since the mechanisms of these two effects are quite different, we believe that the overall effect of CCM will be nontrivially dependent on the precise stimulation parameters. Here, we have identified that the strongest acute positive inotropic effect of CCM on isolated myocytes occurs on the first beat, and then decreases rapidly to be negative. This result suggests that to optimize the effect of CCM on intracellular calcium, one might want to program the stimulator to include pauses between beats for which CCM is turned on. However, this may not be optimal for stimulating the cardiac adrenergic nervous system. A careful optimization of effects of CCM on the cardiac nervous system and cellular contraction may improve its therapeutic outcome.

## Conflict of Interest

None declared.
